# Developing a persona-driven, mHealth-enabled care model for young men with hypogonadism: a methods-based approach to early risk stratification and prevention

**DOI:** 10.3389/frph.2025.1691309

**Published:** 2026-01-22

**Authors:** Francesco Garifalos, Davide Menafra, Cristina de Angelis, Angelica Larocca, Michele Virgolesi, Sara Aprano, Vincenzo De Luca, Teresa Rea, Erminia Attaianese, Mariangela Perillo, Daniela Lemmo, Maria Francesca Freda, Guido Iaccarino, Maddalena Illario, Annamaria Colao, Rosario Pivonello

**Affiliations:** 1 Dipartimento di Sanità Pubblica, Università Degli Studi di Napoli Federico II, Napoli, Italy; 2 Dipartimento di Medicina Clinica e Chirurgia, Università Degli Studi di Napoli Federico II, Napoli, Italy; 3 Dipartimento di Architettura, Università Degli Studi di Napoli Federico II, Napoli, Italy; 4 Dipartimento di Studi Umanistici, Università Degli Studi di Napoli Federico II, Napoli, Italy

**Keywords:** blueprint persona methodology, chronic disease management, digital health, early prevention, hypogonadism, persona-Centered care, unmet needs

## Abstract

**Background:**

Early-onset hypogonadism is an emerging cardiometabolic risk marker in young men, yet current care pathways remain fragmented and insufficiently tailored to patients' multidimensional needs. Digital health technologies offer an opportunity to support early risk stratification, promote self-management, and facilitate long-term prevention. This study presents a methodological framework combining Blueprint Persona modeling and multidisciplinary co-design to support the development of tailored digital health interventions.

**Objective:**

To apply a persona-based, co-design methodology to develop a patient-centered, mHealth-enabled care model for young men with hypogonadism.

**Methods:**

We screened 800 males aged 15–35 years; 55 were diagnosed with hypogonadism. Using the European Commission “Blueprint Persona” methodology, we constructed a representative persona (“Luigi”) and validated it through a multidisciplinary Focus Group (11 experts). Unmet needs were identified and translated into key functional requirements for an mHealth-supported chronic care pathway.

**Results:**

The hypogonadal cohort demonstrated a higher metabolic burden compared to eugonadal peers and reported priorities centred on sexual health, mood and musculoskeletal well-being, and prevention of premature cardiovascular risk. Despite significant psychosocial vulnerabilities, digital readiness was high. The co-design process generated a structured, mHealth-enabled intervention concept integrating teleconsultations, guided diagnostic/therapeutic steps, behavioral monitoring, and adherence-support features.

**Conclusions:**

The Blueprint Persona methodology enabled the systematic translation of clinical, psychosocial, and behavioral insights into a digitally supported care model tailored to young men with hypogonadism. This Methods Article describes the development framework underlying the proposed intervention. A prospective evaluation will assess feasibility, adherence, and impact on cardiometabolic risk and quality of life.

## 1 Introduction

Cardiovascular diseases (CVD) remain a major cause of morbidity and mortality in western countries (1, 2), with 422.7 million cases and 17.92 million deaths reported worldwide (2). Although men show higher CVD prevalence (2), several middle- and high-income countries have documented a decline in CVD incidence and mortality for both sexes (2). However, in the past two decades an increasing or unchanged trend of CVD in young adults—particularly heart failure—has emerged from cohort studies (3–6), while evidence on ischemic heart disease remains limited (7). This pattern likely reflects the growing burden of cardiovascular risk (CVR) factors in this age group, including obesity and unhealthy lifestyle habits such as smoking, physical inactivity, and poor diet, which contribute to early blood pressure abnormalities and metabolic deterioration (8).

A large body of observational and interventional research in middle-aged and older men has demonstrated that metabolic CVR (mCVR) factors—namely obesity, metabolic syndrome (MetS), and type-2 diabetes mellitus (T2-DM) (9–11)—and overt CVD (12–14) are closely associated with low serum testosterone levels, while testosterone deficiency is highly prevalent in obesity, MetS, and T2-DM (10, 15, 16). Cross-sectional and prospective studies have consistently shown that men with total testosterone (TT), calculated free testosterone (cFT), or sex hormone-binding globulin (SHBG) in the lowest quartile present a higher risk of MetS and T2-DM than those with higher levels (17–20), and that men with MetS or T2-DM—particularly in the presence of obesity—display significantly reduced TT, cFT, and SHBG, with negative correlations to BMI, insulin, glucose, and triglycerides and positive correlations to HDL-cholesterol (21–24). Interventional studies on testosterone replacement therapy (TRT) in hypogonadal men with obesity (25–31) and/or MetS or T2-DM (32–34) confirm improvements in anthropometric, lipid, and glycaemic parameters, reinforcing the association between testosterone deficiency and metabolic CVR factors (10). Multiple mechanisms may mediate this interplay, including increased visceral adipose tissue and insulin resistance (IR), cytokine-mediated reductions in SHBG, greater aromatization of free testosterone to estradiol, and impaired gonadotropin secretion driven by inflammation, leptin resistance, and IR (10).

Although evidence in young men is limited, available observational studies suggest that higher TT, cFT, and SHBG levels correlate with a lower prevalence of mCVR factors (35, 36). Given the rising metabolic vulnerability of young adults and the increasing CVD burden in this population, more focused investigation is needed to guide preventive strategies (35, 36). Digital health solutions—such as mobile applications, telemonitoring, and shared care platforms—may support the management of hypogonadism by improving adherence, enabling remote monitoring, and offering psychological support. Evidence from adolescent weight-control interventions (37) and digital care models for Burning Mouth Syndrome (38) suggests that such tools can effectively address these needs.

This approach aligns with the European Blueprint methodology for person-centered digital health interventions, which provides a validated framework for identifying unmet needs and co-designing tailored technological solutions (39). Using this methodology, the present study aims to translate the clinical and psychosocial profile of young hypogonadal men into an actionable, digitally enabled care model supporting prevention and long-term health. Finally, this observational cohort study examines the relationship between androgenic status and mCVR factors in a large sample of young men from the general population, assessing whether testosterone deficiency is linked to early dysmetabolic conditions and may predict future CVD.

## 2 Materials and methods

This study adopted a mixed qualitative/quantitative approach based on the development of a person-case using the “Blueprint” methodology. This framework captures behavioral characteristics to account for the psychosocial forces and health-related choices that may influence outcomes which could be improved through the adoption of digital solutions. Personas are used to describe unmet needs and potential IT solutions, identifying enabling technologies and high-impact use-case scenarios (39). A persona is defined as a single, specific hypothetical individual representing a segment of the population, described through a realistic name, a face, and a characterization that includes needs, goals, motivations, and attitudes. Blueprint personas also incorporate behavioral elements that may affect short- or long-term success with interventions, such as trust in healthcare professionals, self-management skills, or a tendency to resist external support.

In this study, a collaborative approach was used to identify a blueprint persona named Luigi, a 33-year-old man. Several characteristics were associated with this persona, including clinical profile, lifestyle information, social context, technological readiness, and unmet needs relevant to patient management. The survey used to inform the persona was administered anonymously, and no approval from the Medical Ethical Committee was required.

The theoretical elaboration of prototypes representative of hypogonadal patients was developed through an interdisciplinary Focus Group including:

- 3 andrologists/endocrinologists
- 3 residents in endocrinology
- 1 psychologist
- 2 experts in architectural solutions/architects
- 1 nurse
- 1 expert in digital health
- 1 nutritionist

### 2.1 Subjects' recruitment

A total of 800 male subjects, aged 15–35 years, were recruited at the Andrology Clinic of the FERTISEXCARES Center, Department of Clinical Medicine and Surgery, AOU Federico II of Naples, as part of a screening campaign aimed at the prevention of andrological diseases in young adults and the early identification of hypogonadism as a potential predictor of future metabolic syndrome and cardiovascular diseases.

Exclusion criteria included the presence of psychiatric comorbidities that could impair comprehension of the study protocol, substance abuse or alcoholism, poor compliance, and the use of psychotropic medications.

### 2.2 Focus group validation

To validate and refine the persona, an interdisciplinary Focus Group (*n* = 11 experts) was convened and conducted over four structured sessions held between September 2023 and March 2024. Using the Blueprint Persona Development Tool, participants:

1. Reviewed screening data from hypogonadal patients recruited into the study.
2. Mapped Luigi's behavioral and clinical traits through iterative group discussions.
3. Prioritized unmet needs using anonymous Likert-scale voting (1–5).
4. Proposed digital solutions aligned with existing hospital services.

Consensus was defined as agreement by ≥80% of participants regarding persona accuracy and need prioritization. Audio recordings from the sessions were analyzed thematically using NVivo 12.0. Based on this qualitative synthesis, clinical and psychosocial characteristics of hypogonadal patients were integrated to construct a prototype persona—Luigi—representing the most frequent profile observed (a 33-year-old man with obesity, depressive symptoms, and challenges in therapy adherence).

### 2.3 Blueprint persona elicitation and digital solution

Based on Focus Group consensus, a theoretical elaboration of the Blueprint persona was developed, identifying three major unmet needs: (1) social isolation, (2) therapy adherence, and (3) sexual health. The digital health team then translated these needs into an applicable solution by integrating:

- Telemedicine features already available at Federico II University Hospital
- Behavioral nudges derived from evidence-based mHealth frameworks
- Architectural recommendations to support adaptations in home and workplace environments

## 3 Results

The screening cohort included 800 participants (mean age 32.7 years; range 15–35), among whom 55 met diagnostic criteria for hypogonadism (6.8% prevalence). As summarized in Table 1, the hypogonadal subgroup exhibited a marked metabolic burden: 43% (*n* = 24) were overweight, 27% (*n* = 15) obese, 23% (*n* = 13) hypertensive, and 27% (*n* = 15) had dyslipidemia. Regarding lifestyle factors, 34% (*n* = 19) were active smokers, while excessive alcohol consumption was reported by 7% (*n* = 4). In comparison, among the 745 eugonadal subjects, the prevalence of overweight and obesity was 36% (*n* = 268) and 18% (*n* = 134), respectively; 10% (*n* = 75) presented hypertension, and 18% (*n* = 135) had dyslipidemia. These findings indicate that young hypogonadal men exhibit a markedly higher metabolic burden than their eugonadal peers, reinforcing the relevance of early identification and tailored clinical management.

**Table 1:** Demographic, clinical, and psychosocial characteristics of the hypogonadal subgroup (*n* = 55). Data are presented as percentages (n) or mean values [range].

Variabile	Valori (%) o [media ± DS]
Età	32.7 anni [range 15–35]
Livello di istruzione	
Primaria	11% (*n* = 6)
Secondaria	57% (*n* = 31)
Universitaria	32% (*n* = 18)
Area di residenza	
Urbana	37% (*n* = 20)
Suburbana	56% (*n* = 31)
Rurale	7% (*n* = 4)
Comorbidità	
Sovrappeso	43% (*n* = 24)
Obesità	27% (*n* = 15)
Ipertensione	23% (*n* = 13)
Dislipidemia	27% (*n* = 15)
Stile di vita	
Fumatori attivi	34% (*n* = 19)
Consumo elevato di alcol	7% (*n* = 4)
Occupazione	
Occupati	38% (*n* = 21)
Disoccupati	62% (*n* = 34)
Vita sociale	
Attiva	47% (*n* = 26)
Povera	53% (*n* = 29)

Psychosocial evaluation revealed substantial socioeconomic challenges, with unemployment documented in 62% (*n* = 34) of subjects and limited social engagement in 53% (*n* = 29). Despite these vulnerabilities, digital readiness was notably high: nearly all participants owned a smartphone or tablet (99%, *n* = 54) and had broadband access (97%, *n* = 53). In addition, 95% (*n* = 52) reported access to technical support from family members or caregivers.

Patient-reported outcome measures consistently highlighted three major therapeutic priorities: (1) restoration of sexual function, (2) improvement of musculoskeletal health and affective state, and (3) mitigation of premature cardiovascular risk. These priorities informed the conceptualization of the proposed mHealth intervention.

### 3.1 Male hypogonadism persona profile

The persona *Luigi*, derived from cohort data and refined through the Focus Group process, represents the most recurrent clinical and psychosocial features observed among young men with hypogonadism (Table 2). Luigi is a 33-year-old man living with his wife in a suburban area of Naples. His clinical profile includes hypogonadism, obesity, osteopenia, and comorbid depressive symptoms, accompanied by a sedentary lifestyle with minimal structured physical activity. His leisure time is largely devoted to passive activities such as watching television or reading magazines, and he reports poor sleep quality, sexual dissatisfaction, and suboptimal adherence to prescribed therapies. He consumes alcohol occasionally but does not smoke.

**Table 2:** Persona profile – blueprint analysis framework. This template provides a structured overview of life domains, contextual factors, and unmet needs, enabling focused discussion in workshops and supporting the design of human-centered health and social services.

PROFILE OF: LUIGI (AGE: 33)
LIFE COURSE: Working age adults
PRIMARY NEEDS: Social connection & Health self-management
PROFILE SUMMARY:
Luigi is a 33-year-old man navigating multiple complex health and social challenges. His situation is significantly compounded by an unsupportive living environment, which actively exacerbates his health conditions and severely limits his opportunities for social engagement. Although he possesses the basic technical means, including access to technology and devices, a critical lack of digital health literacy prevents him from effectively managing his own care and leveraging these tools to his advantage KEY PRIORITIES (What's Important to Luigi): - Improve sexual health and activity - Enhance mood and muscle strength - Prevent premature death - Develop motivation for self-care and treatment adherence ENVIRONMENTAL BARRIERS & RESOURCES: - Residential: Lives with wife in suburban Naples; lacks parks/green spaces - Neighborhood: Heavy traffic; unsafe footpaths; poor community facilities - Housing: 5th floor apartment with no elevator; 5 steps at building entrance - Home Environment: Poor lighting; traffic noise; slippery floors; tripping hazards - Transportation: Infrequent public transport DAILY LIVING PATTERNS: - Reduced mobility and functional ability - Sedentary lifestyle dominated by work - Leisure time spent watching TV and reading magazines - Limited physical activity and social engagement HEALTH STATUS CONDITIONS: - Hypogonadism - Osteopenia - Depression - Abdominal obesity RECENT HEALTH TESTS: - Andrological examination - Cardiological screening with Doppler ultrasound - Bone density scan (MOC-DEXA) - Blood pressure monitoring and blood tests TREATMENT REGIMEN: - Currently prescribed 3 different medications - Demonstrates poor treatment compliance PERSONAL CONCERNS & CHALLENGES: - Poor sleep quality - Depression and sexual dissatisfaction - Difficulty maintaining motivation for treatment - Resistance to change due to feelings of helplessness - Low confidence in self-care abilities CLINICAL CONCERNS (Care Team Perspective): - Poor therapy compliance - Inability to adopt healthier lifestyle behaviors - Need for comprehensive support strategy SOCIAL & EMPLOYMENT CONSIDERATIONS: - Multidimensional approach needed for social integration - Active lifestyle promotion required - Psychological support could improve work engagement EDUCATIONAL NEEDS: - Integrated nutritional education - Psychological support strategies - Parallel education with medical therapy TECHNOLOGY PROFILE: - Broadband access: Yes - Smartphone/tablet access: Yes - Internet comfort: Yes - Device comfort: Yes - Willingness to learn new tech: Yes - Digital health literacy: Low (0) - Technical support available: Yes RECOMMENDED TECHNOLOGY SOLUTIONS: 1. Integrated monitoring app with: - Outcome notifications - Appointment calendaring - Clinical progress tracking 2. Telehealth services: - Remote monitoring - Online consultations 3. Self-care motivation app featuring: - Personalized notifications - Multidisciplinary team access - Outcome tracking 4. Online motivational interviews for: - Long-term benefit visualization - Self-care motivation development 5. Community connection platform for: - Peer support - Reduced isolation UNMET NEEDS: - Social connection and relationships - Sexual health management - Mood regulation and depression treatment - Self-care motivation and lifestyle modification - Polypharmacy and treatment adherence support - Successful aging planning RECOMMENDED APPROACH: Comprehensive care plan integrating: - Medical treatment optimization - Environmental modifications - Technology-enabled support - Social engagement opportunities - Psychological and nutritional support

The Focus Group highlighted several unmet needs commonly exhibited by patients with similar profiles, particularly in the areas of social integration, sexual health, therapeutic adherence, and mood regulation. These domains were identified as priority targets for tailored mHealth interventions, as they offer opportunities for structured digital support, remote monitoring, and behaviorally informed engagement strategies.

**Figure 1:**
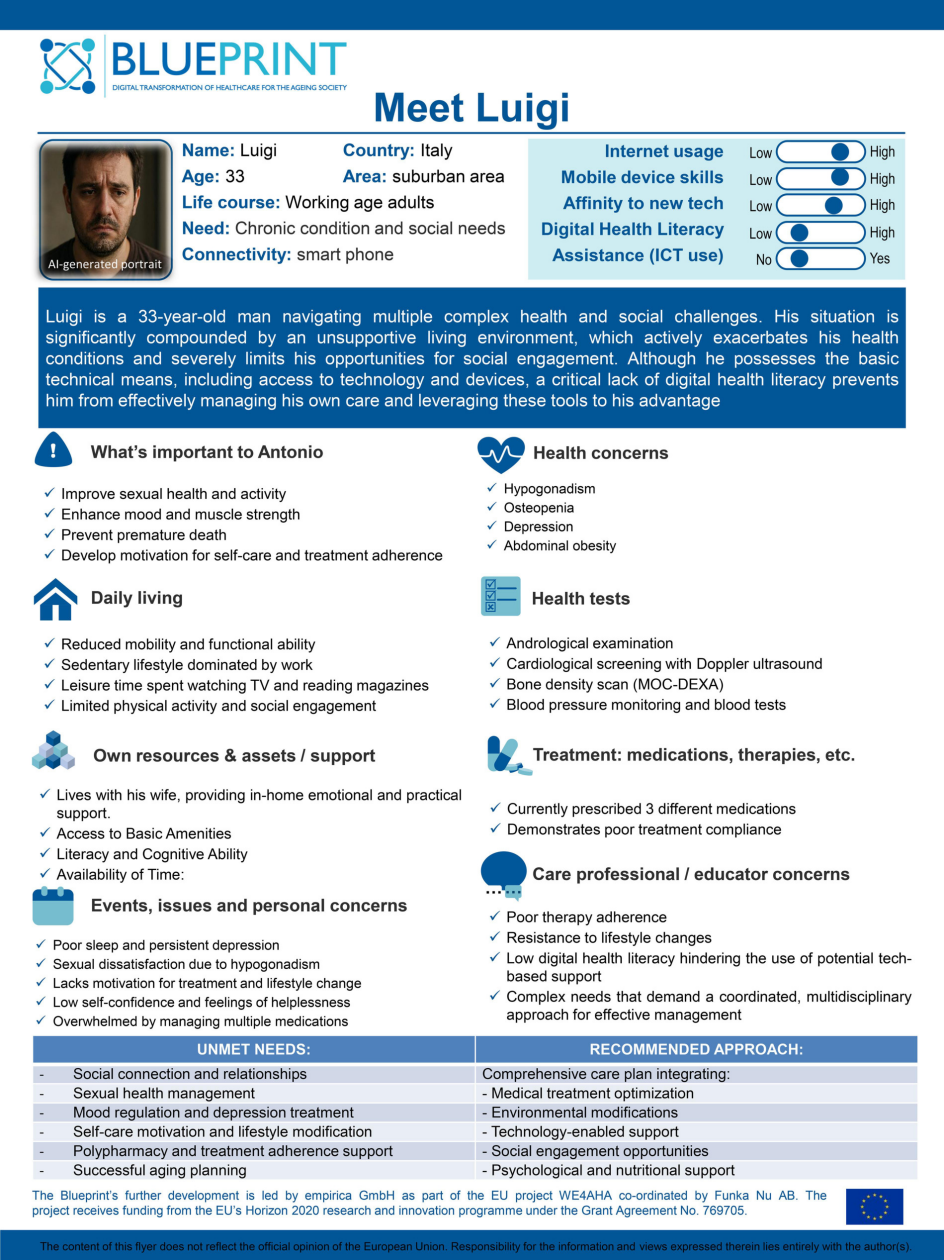
Patient profile blueprint poster: this visual tool enables multidisciplinary teams to rapidly 272 identify complex patient needs and co-design targeted interventions. It synthesizes medical, social, and environmental 273 factors into a holistic overview, aligning with blueprint methodology to ensure actionable and human-centered care 274 planning.

To visually synthesize Luigi's complex clinical and psychosocial profile, a Blueprint persona poster was developed (Figure 1). This tool serves as a shared reference for multidisciplinary teams, allowing rapid identification of interconnected needs and supporting the co-design of personalized interventions.

Within this framework, nurses—integral members of the multidisciplinary team—play a crucial role in addressing the needs of patients with hypogonadism. By establishing trusting relationships, they help improve treatment adherence, mitigate the impact of symptoms, and contribute to the prevention of long-term cardiovascular complications (40, 41). Nurses act as coordinators across healthcare settings, providing continuous support to patients as they navigate diagnostic and therapeutic pathways (42). They address both overt and hidden needs through systematic symptom assessment and hormonal monitoring, delivering essential education on the condition and treatment options. Moreover, they integrate mHealth tools—such as health management apps and telemonitoring systems—to promote proactive symptom tracking and encourage healthier routines (43–45). Management of hormone replacement therapy includes clear explanations of benefits and potential side effects, along with ongoing reassessment of the patient's status (46).

Nurses work in close collaboration with other professionals to manage not only the physical manifestations of hypogonadism but also relational and psychosocial challenges, providing a comprehensive response to patients' needs. This holistic approach is particularly valuable for complex patients like Luigi, who may encounter barriers to self-management and adherence due to the multifaceted nature of their condition (47).

In this persona profile, the clinical psychologist plays a key role in strengthening self-care motivation by identifying areas of resistance to change and difficulties in adapting to illness and engaging with healthcare. According to the Illness Representation Model, a patient's interpretation of their condition directly influences their propensity to adopt self-care behaviors (48). Recent studies indicate that patients with hypogonadism often perceive their condition as chronic, burdensome, and linked to reductions in mood, sexual satisfaction, relationship dynamics (48), and overall quality of life (49, 50). Such perceptions may lead to helplessness and reduced motivation to maintain long-term treatments. Self-efficacy—confidence in one's ability to manage the disease—is another determinant of self-care: patients with hypogonadism frequently underestimate their ability to control their condition, making them less likely to adopt active strategies such as lifestyle improvements or medication adherence (51, 52).

Within the multidisciplinary team, the psychologist contributes to prevention and health promotion by addressing behavioral risk factors and strengthening motivation for change. Analyzing motivational processes helps identify barriers to new behaviors and provides tools for overcoming them. For instance, resistance to change can be addressed through motivational interviewing, a collaborative approach aimed at enhancing autonomy and confidence. Digital tools and behavioral monitoring may offer additional support, increasing awareness of progress and reinforcing the adoption of healthier routines, thereby improving engagement and motivation.

The nutritionist's role is essential within this multidimensional framework, as proper nutritional management is a key component in the care of patients with hypogonadism. Numerous studies indicate that obesity is strongly associated with hypogonadism (53), particularly of the secondary type, and that adherence to a Mediterranean diet may contribute to restoring eugonadism (54).

Environmental factors also exert a significant influence on individuals with hypogonadism. Research shows that living in low-income areas—characterized by inadequate land use, exposure to neighborhood stressors, and limited access to healthcare resources or beneficial amenities such as parks—may worsen outcomes, as these conditions promote stress, reduce physical activity, and hinder preventive care (55, 56). Hypogonadism has also been linked to oxidative stress induced by environmental pollutants, including pesticides, radiation, air pollution, heavy metals, and other endocrine-disrupting chemicals (57). Exposure occurs through multiple pathways: pesticides can be ingested via contaminated foods, with higher residue levels associated with reduced sperm count; radiation exposure is widespread due to devices such as laptops, cell phones, microwave ovens, Wi-Fi networks, radar systems, and medical equipment including x-ray and radiotherapy devices (58–60). These pollutants—especially pesticides (chlorpyrifos, diazinon, cypermethrin), radiation sources (EBRT, mobile phones), heavy metals (cadmium, lead), and plastic-derived endocrine disruptors (e.g., BPA)—can disrupt oxidative balance, leading to impaired sperm viability and motility, alterations in ionic homeostasis, and significant changes in the sperm proteome (61).

### 3.2 Applicable digital health solution

The digital solution proposed consists of a mobile application designed to serve as an accessible tool supporting the needs identified in the persona profile. The app would provide a user-friendly interface enabling patients to address several unmet needs highlighted during persona development. Specifically, it would allow patients to conduct telemedicine assessments with the multidisciplinary team involved in their outpatient care. The application would also help patients schedule clinical appointments, integrating reminders into a personalized agenda to improve compliance and reduce missed follow-ups. In addition, the app would support positive reinforcement strategies by monitoring clinical parameters and biochemical improvements, offering motivational feedback over time.

To achieve this, dedicated digital modules will be incorporated into the app, enabling both patients and clinicians to record relevant clinical data. The platform will also include links to validated questionnaires and assessment tools designed to evaluate sexological and psychological progress.

Given the cohort's high prevalence of sedentary behavior (53%, *n* = 29) and obesity (27%, *n* = 15), integrated adapted physical activity (APA) interventions are essential. Personalized APA programs—delivered through web platforms featuring video tutorials and communication channels for interaction with clinicians—may help address exercise resistance while accounting for socioeconomic limitations such as unemployment (62%). Wearable monitoring tools (e.g., step counters, activity intensity, vital signs) and virtual reality systems may further enhance engagement, particularly among patients with depressive symptoms (44%) or limited social support (53%).

From a psychological perspective, identifying patterns of motivational resistance is fundamental. Fear of failure, low perceived self-efficacy, and insufficient emotional support often hinder behavioral change. Targeted interventions that provide tools for tracking self-care progress may therefore offer significant assistance to patients like Luigi.

## 4 Discussion

The increasing prevalence of acquired hypogonadism among young men represents a major health concern, as this condition—often secondary to metabolic disorders—creates a bidirectional relationship with obesity, dyslipidemia, and diabetes. Early-onset hypogonadism may therefore accelerate the transition from successful to pathological ageing, with long-term cardiometabolic and psychosocial consequences. Beyond its physical manifestations, hypogonadism is strongly associated with reduced mood, impaired sexual health, social withdrawal, and poor adherence to therapy, all of which negatively affect quality of life.

Our findings, supported by the Focus Group analysis, indicate that complex patients such as Luigi present multiple unmet needs that require integrated and personalized care models. Traditional, episodic approaches often struggle to address needs spanning social integration, sexual health, and therapeutic adherence. This gap underscores the relevance of continuous, coordinated, and person-centered strategies, such as those enabled by our proposed mHealth platform. Digital health tools have demonstrated growing utility in this context. In sexual medicine, recent studies report high levels of patient satisfaction with telemedicine consultations, emphasizing their ability to improve accessibility and continuity of care for sexual dysfunction management (62). Similarly, mobile health devices have shown feasibility in monitoring sleep disorders, which are frequently reported by men with hypogonadism (63). International expert consensus documents further highlight the role of telemedicine in men's health, recommending its integration into diagnostic and follow-up pathways (64).

Lifestyle modification also remains central in the management of young men with hypogonadism. Aerobic exercise has been shown to attenuate obesity-related hypogonadism by improving body composition and metabolic function (65). In this context, structured Adapted Physical Activity (APA) programs may represent an effective non-pharmacological strategy to counteract sedentary behavior, promote hormonal balance, and support psychological well-being. Integrating such programs within digitally monitored environments may further enhance adherence and sustainability.

From a clinical standpoint, a multidisciplinary approach involving endocrinologists, psychologists, nurses, nutritionists, and digital health experts is essential to address both evident and hidden needs. Nurses, in particular, contribute substantially by supporting adherence, educating patients regarding hormone replacement therapy, and bridging communication across care settings—roles that become even more central when digital tools are integrated into routine practice (41–47).

A key innovation of this study lies in the application of the Blueprint Persona methodology, which provided a structured, patient-centered approach to move beyond clinical biomarkers and understand the broader lived experience of patients. This framework facilitated the identification of Luigi's unmet needs and their translation into actionable digital and behavioral interventions. Future interventional studies will be necessary to assess the feasibility, clinical impact, and acceptability of such integrated solutions, with specific attention to adherence, clinical outcomes, and quality-of-life improvements.

Ensuring long-term sustainability of digital health interventions requires their integration into existing care pathways, alignment with reimbursement structures, and demonstration of cost-effectiveness. Embedding the mHealth platform within routine organizational workflows may help optimize resource allocation and reduce the long-term burden of untreated cardiometabolic complications (66).

As with all digital interventions, ethical considerations related to data privacy, patient autonomy, and the potential risk of stigma must be carefully addressed. Secure data governance and equitable access are essential to ensure that digital tools strengthen—rather than undermine—patient trust and engagement (67).

Although the persona-based approach was developed specifically for young men with hypogonadism, the methodology is inherently replicable and could be adapted to other chronic conditions characterized by complex unmet needs. By tailoring this framework, similar interventions may support prevention and self-management across diverse patient populations (37, 38, 68).

## 5 Conclusions

In conclusion, this study demonstrates the utility of the Blueprint Persona methodology as a foundational framework for co-designing patient-centered digital health interventions. By moving beyond purely clinical phenotypes to incorporate the holistic lived experience of patients—exemplified by the persona “Luigi”—we translated complex unmet needs into a tailored, feasible, and comprehensive mHealth-enabled care model for young-onset hypogonadism. This approach effectively operationalizes early risk stratification and prevention within a chronic disease management framework, addressing a critical gap in current care pathways.

The proposed strategy aims not only to improve clinical outcomes but also to strengthen patient empowerment through sustained self-management. Future research should focus on prospectively evaluating this integrated solution to determine its impact on treatment adherence, cardiometabolic risk profiles, and quality of life, thereby supporting its potential implementation in routine clinical practice. Importantly, the persona-based methodology presented here is inherently scalable and replicable, offering a validated blueprint for addressing the multifaceted needs of patients living with other chronic conditions.

## Data Availability

The original contributions presented in the study are included in the article/Supplementary Material, further inquiries can be directed to the corresponding author
